# Supplementary material for: The internal realities of individuals with type 2 diabetes–Psychological disposition in self-management behaviour via grounded theory approach
Source: PLoS One. 2021 Apr 13;16(4):e0249620. doi: 10.1371/journal.pone.0249620 (PMC8043383; doi:10.1371/journal.pone.0249620)
Supplement: S1 File — (DOCX) [file pone.0249620.s001.docx]

**S1 File.**

**TOPIC GUIDE (In-Depth Interviews)**

**Initial Open Ended Questions**

1. What do you know about diabetes as a disease?
2. How long have you been a diabetic patient? How do you think is your sugar control so far?
3. Can you tell me what is like being a diabetic patient? What do you feel being called a diabetic?
4. What do you feel about your control over your diabetes as a disease? Are you confident in controlling your disease? Why?
5. How did you feel when you were told you need to take long term medications?
6. Could you describe what do you think about diabetic medications?
7. You have been taking your medications for some time. Can you describe your experience taking medications?

- What do you dislike about taking medications?
- What are the benefits of taking medicine and not taking medicine?

1. Could you explain to me your concerns about taking medications?
2. How is your relationship with your doctor in terms of taking medication?

- Do you trust what is prescribed to you by your doctor? Do you seek advice of other doctors?

**Intermediate Questions**

1. Could you tell me how you seek information on how to improve the way you take your medicine?
2. Do you believe you have sufficient information to adhere to medication compliance?
3. Explain to me how you take your medication daily? How has your doctor advised you to take your current medications? Do you think you are taking it the right way?
4. What do you think about the importance of taking your medication?
5. What are the problems you face in order to take your medications properly?

- What makes it easy for you take medications as advised?
- What makes it difficult to take medications as advised?

1. How do you overcome these problems that cause you to miss your medications?
2. In general, do you feel that you take your medication on a regular basis?
3. Some patients say it is difficult for them to take medications daily. What is your opinion on this? How much effort does it require take your medication?
4. Explain to me what you know about the complications of diabetes?
5. Do you think you are invulnerable to the complications of diabetes? If no/yes, then why?
6. What do you feel are the chances of you developing complications from diabetes in the future if you don’t take your medication?

**Ending Questions**

1. What do you think is the most important way to be compliant to medications?
2. What do you think is your ability to find solutions and solve problems related to diabetes?
3. How do you think is the control of diabetes in other patients in general, poor or good?
4. What do you think is your role in ensuring proper intake of your medication other than healthcare providers?
5. How have you grown as a person since you were diagnosed with diabetes and the need to be reliant on medications?
6. Is there anything else you think I should understand about your medication compliance better?
7. Is there anything else you would like to ask me?

**EMERGENT THEME 12^TH^ ITERATION (In-Depth Interviews)**

1. Insulin versus OHA

- What do you think about taking insulin? What do you think about taking insulin in front of others?

- What do you think others will think?

2. Body sensation/‘physical’ feeling of being well

- How does your overall wellbeing influence your intake of medications?

- How does feeling well bodily influence your intake of medication?

- How is your medication intake when you feel unwell?

3. Acute versus chronic mismatch

- There are some diseases for example fever/gout that can cause immediate pain, what are your thoughts about

diabetes in relation to this?

- How do you think diabetes be cured? Quick fix?

- How does this thinking influence your medication intake?

4. Family support

- How importance is the role of family in compliance with medication?

- How do your family members feel responsible for your health and intake of medication?

- How do they ensure you take your medication?

5. Patient centeredness

- How would define or explain your consultation with your doctor?

- How do you think the relationship with your doctor influences your medication intake?

- How do you think seeing the same doctor affects your intake of medication?

- Do doctors understand your medication intake? How should do you expect them to help you?

6. Working environment/house hold environment

- How does your working environment/schedule affect medication intake?

- How do the other activities in your life (family) influence your intake of medications?

- How can you make changes to your work schedule/chores to prevent missing taking your medication?

- Which part of the day do you miss your medication? Why?

7. Food and exercise can control disease better than drugs?

- What do you think about is the importance of diet and food in the control of diabetes?

- How do you think food and exercise control can replace your medication?

8. Self efficacy

- Is there anything that could make you more responsible in taking your medication?

- What steps would you take so that you would always take your medication?

- If you want to know you are doing the right thing to control your diabetes, is there anything you would do to

confirm that you are doing the right thing?

- Do you read the label on your prescription? Why not?

- Do you check for your sugar levels at home? What made you do it?

9. Traditional medication

- What do you think about traditional medication?

- How does taking traditional medication affect your compliance to diabetic medications?

- What do you feel about combining these two types of treatment?

10. Feeling restricted

- You were probably told to avoid food and maintain a strict lifestyle, do you ever feel restricted? Why?

11. Taking medication is a sign of weakness/feel like sick person

- Some patients say it’s a sign of weakness, taking meds. What do you think about this?

- Some patients say taking medication makes them feel like a sick person? What do you think?

12. Side effects of medications

- What are the side effects your experience? What are they side effects you fear?

- How does this side effect affect your medication intake?

13. Experimentation with medication

- Doctors have written and give you advice on how to take your medication but there are people who experiment

with their drug dose or regime? What do you think about this? Does this happen to you?

14. Healthcare system

- How would you rate the public healthcare system in terms of diabetes management?

- How do you think the quality of care influences your intake of medications?

15. Education

- What do you think is the role of education in staying compliant with medication?

- What do you think is the difference in taking medication in highly educated and average educated people?

16. Forgetfulness

- Some patients say they forget to take their medications. Why do you think that happens in your case?

- There are some patients who believe that if we can remember to eat our food, why do we forget to take

medications? What’s your opinion?

17. Fear of uncertainty of the disease

- Some patients say that they are uncertain of the disease, there can be ups and downs, and circumstances can

change anytime. What are your thoughts on this?

18. Skip taking medication

- Some patients skip taking medication? Does that happen in you? Why does that happen?

19. The efficacy of medication

- Do you think the effect of medication is subjective, or the same for all patients? Some patients think the drug

works slower in the compared to others. What’s your opinion?

20. Age

- Does age influence your intake of medication? How? What was your behaviour when younger and now? How

does it compare?

21. Culture of diabetes

- Do you think diabetes is a norm in our society? Why?

- Compared to other diseases (for example smoking), what do you think of diabetes? What do you feel like

living with diabetes in the community?

- What do you feel/your reaction when you find out that a close friend/relative has been newly diagnosed?

22. Conflicting thoughts about medication (dissonance)

- Do you get conflicting thoughts about taking and not taking your medication? Why, please explain?

23. Feel that there is a need to take less/reduce medication?

- Do you feel you experiment because you want to take less medication?

**TOPIC GUIDE (Focus Group Discussion)**

**Main Questions 1**

- Can you please explain what external factors that exert control over the management (compliance) of your disease?
- What are your thoughts about fixed factors that you cannot control?

**Stems (Emerging Theories)**

- What do you think about your food environment? How do you think that influences T2D control? Do you think there needs to be proper legislation?
- How has your lifestyle influenced your medication intake (work, compliance, insulin vs medication), diet and exercise?
- How does your social circle influence you? How about social pressure? Does it lead to dietary violations?
- How expensive do you think is it to manage your disease? Why?
- What do you think is the influence of religion? Do you think it helps you manage disease better?
- What is the influence of age, education, financial status on T2D control/compliance?
- How do you think your relationship with your doctor and other T2D patients (sharing information) affects your compliance?
- What is your opinion about the service at the clinic or by HCP? How do you think it can be improved?
- How does work influence your medication intake?
- How have other people (similar) with T2D influenced the way you view your disease?
- Do you have any limitation now that you have T2D? What are they?
- Do experience any physical symptoms that alert you that something is wrong with your control (uncontrolled, missing medication, disagree so comply to medication, monitoring based on symptoms, no influence)? Explain. [Physical]
- What are the issues that make you uncertain your control of the disease (uncertain about nature of disease, suspicious about glucometer, insulin ineffective, cannot control disease)?
- After being commenced on medication, how do you feel (physically better, insulin better, CAM good control)?

**Main Questions 2**

- How about your personal experience about taking medication and the disease?
- What are your our thoughts about managing the disease?

**Stems (Emerging Theories)**

- What is your experience taking medication all these years (timing, side effect, reduce dose)?
- How about influence of others affected the way you take medication (discouraged, opinion of others)?
- What negative events have you experienced (side effect, physical, complications, problems with medications? How has that influenced you?
- Have you wondered about how other people deal with T2D (healthy despite unhealthy, different opinion from doctor, some people unaffected)

**Main Questions 3**

- What about your general world view (ideas from others and yourself) of the disease?
- What are you inner opinion about the yourself and the disease?

**Stems (Emerging Theories)**

- What is your perception of the control in yourself and others? What is your opinion about diabetes in this country?
- What do you believe about the effects of medication (taking too much, insulin and oral, kidney, ill health), CAM (natural sources), food (rice), exercise?
- What causes T2D, complications, where medication is processed?
- What are your expectations? What do you want from doctors (same doctor, two way, empathy, reduce dose) or family members?
- What factors do you blame for poor control? Who do you blame for poor control? Does having T2D affect you in terms of productivity or inability to work?
