# Supplementary material for: The internal realities of individuals with type 2 diabetes–Psychological disposition in self-management behaviour via grounded theory approach
Source: PLoS One. 2021 Apr 13;16(4):e0249620. doi: 10.1371/journal.pone.0249620 (PMC8043383; doi:10.1371/journal.pone.0249620)
Supplement: S1 Fig — (DOCX) [file pone.0249620.s002.docx]

***Triangulation 1***

***Emergent themes from In-Depth Interviews (IDI)***

A review of scientific literature was conducted to identify areas that define self-management activities

A preliminary topic guide was designed and emergent themes were elicited via Ground Theory Approach

After the 10^th^ interview, no additional themes were discovered and theoretical saturation was considered to have been reached

The topic guide was revised

***Concurrent revision of topic guide***

***Methodological Triangulation***

A preliminary topic guide was designed and emergent themes were elicited via Ground Theory Approach from individuals with diabetes

After the 10^th^ interview with healthcare providers, no additional themes were discovered and theoretical saturation was considered to have been reached **(negative case analysis)**

***10^th^ iteration of topic guide***

***Theoretical Sampling (IDI)***

Additional interviews with individuals with diabetes

14 interviews

Emergent themes explored, enriched and consolidated

Development of topic guide for focus group discussions

***Conceptual Framework***

***12^th^ iteration of topic guide***

***Data Triangulation***

Transcribed interviews coded by researcher

Cross discussion with collaborators to establish themes **(persistent observation)**

Inter-coder reliability testing **(investigator triangulation)**

Framing of themes

Codebook generated

Kappa inter-rater reliability/expert consensus

***Focus Group Discussions (FGD)***

Total of 12 individuals with diabetes in 2 sessions

All themes derived from IDIs tested and verified

***Triangulation 2***

**S1 Fig. Flow chart of the study design and development of conceptual framework**
