# Supplementary material for: The internal realities of individuals with type 2 diabetes–Psychological disposition in self-management behaviour via grounded theory approach
Source: PLoS One. 2021 Apr 13;16(4):e0249620. doi: 10.1371/journal.pone.0249620 (PMC8043383; doi:10.1371/journal.pone.0249620)
Supplement: Attachment — Submitted filename: Response To Reviewers.docx [file pone.0249620.s005.docx]

**Response to Reviewers**

We would like to thank the PLOS One to give our paper a second look. We also thanks the reviewers for devoting their time to provide very insightful observations. We are certain this would definitely help strengthen the merit of our paper. Do not hesitate to keep us informed if further revisions are required.

*Comments from Reviewer 1*

1. Line 14: Remove “a” before “T2D patients that were….”

- We accept this suggestion and have made changes accordingly

Page 2, Lines 14:

in order to obtain T2D patients that were representative……..

2. Twenty-four T2D participants and 10 healthcare professionals were recruited to examine the participants’ narratives about disease management and what they believe about the disease.” This sentence is not clear whether who are the participants, so I suggest to rephrase it with “24 T2D patients and 10 healthcare professionals…

- We accept this suggestion and have made changes accordingly

Page 2, Lines 15:

24 T2D patients and 10 healthcare professionals were recruited

3. Line 33: Change “sensitivies” to “sensitivities”

- We agree with this comment and have made changes to the manuscript accordingly

Page 2, Lines 33:

These findings allow the implementation of psychosocial changes that are in line with cultural sensitivities and societal norms.

4. Introduction. Line 26: I suggest you remove “previously published protocol manuscript” and describe the framework

- We concur and made changes to the manuscript as described below.

Page 3, Lines 26:

This paper attempts to implement published procedural methods in a bid to obtain salient qualitative information central to the development of an integrated behavioural model that explains self-management practices in T2D patients

5. Methods - Line 9-10: Remove “previously published protocol” and “A published study protocol of this endeavour provides a more detailed description of the research methods employed here” instead describe the method in brief and provide reference to the published study protocol.

Page 3, Lines 9-10:

Furthermore, the strategy of theoretical sampling was utilized to identify information-rich cases that would help saturation of emergent constructs that explain self-management behaviour in T2D patient.

6. Line 19-20: Remove this sentence “Information on research technicalities and participant recruitment methods are found in previously published article closely associated with this study” and elaborate the method and provide reference.

- We concur and made changes to the manuscript as described below.

Page 4, Lines 19-20:

T2D patients who were recruited in the study had the following characteristics, 1) age above 18 years old, 2) fluent in both English and Malay language, 3) a diagnosis of T2D as documented in their case records, and 4) received followed-up at the clinic for a minimum duration of 2 years[11]. The details about the study was conveyed directly to each patient by the principal investigator during their routine medical consultation at the clinic. Similarly, HCPs comprising of GPs, diabetic educator (nurses) and pharmacists were invited to join the study. After obtaining informed consent, all participants were given an appointment to attend a scheduled interview session.

7. In the data collection section, it is mentioned that “Thirty-four people T2D patients and 14 HCPs were invited to participate in the study”

-Remove “people” before T2D patients in line 4
-Since both are the participants in the study please be consistent throughout the paper mentioning both as participants and maintain consistency in words with “Healthcare professionals and healthcare workers and health care providers” and “T2D patients and T2D participants”. I suggest you write healthcare professionals and T2D patients throughout the paper.

- Thank you for your comment, we have amended the manuscript accordingly.

8. Line 11-12 “A previously published protocol explains details related to interviewing technique and the topic…” remove “previously published protocol” and elaborate the technique with reference.

- Thank you for your comment, we have amended the manuscript accordingly.

Page 6, Lines 11-12:

IDIs were guided by research questions (compiled in the form of a topic guide) designed to assess T2D patient’s inherent psychological attributes that are closely associated with self-management behaviour[11]. Steps were also taken to ensure these interviews remain unstructured enough to facilitate to discovery of novel concepts and ideas. Moreover, the topic guide was constantly updated in tandem with the acquisition of newer themes from these interviews.

9. In data management and analysis section, line 10 “…themes derived from this study was assessed…” change was to were.

- Thank you for your comment, we have amended the manuscript accordingly.

10. The result section needs formatting with separating the words attached with spaces.

- Thank you for your comment, we have amended the manuscript accordingly.

11. There is no full form of the abbreviation “IDM” in the verbatim.
    The verbatim needs to be given demographic details like for example: “35-year-old T2D, self-employed male, FGD participant”

- Thank you for your comment, we have amended the manuscript accordingly.

12. Discussion - The authors mention that there are four constructs and identify two major themes in the discussion (line 30-31) which is not clear.

- We thank the reviewer for pointing this aspect of the journal. We have modified the manuscript to describe what we meant to convey with more accuracy in the results section.

Page 7, Lines 21-28:

The internal psychological reality of T2D patients defining their self-management practices can be summarized into 2 main constructs – 1) positive disposition and 2) negative disposition. There appears to be a dynamic interaction between these constructs and numerous other concepts shown in Figure 1 – the factors associated with the external reality and the mediators of optimal behavior create the tapestry for the workings of the internal realities of T2D patients.

13. Conclusion
    The conclusion that “… the constructs discovered in this study can be quantified in the form of a self-administered questionnaire as described in a recent study protocol” is not clear. Please elaborate and do not direct to the protocol study instead reference it.

- We have amended this part of the manuscript to reflect better accuracy of what we meant to convey.

Page 17, Lines 18-21:

Furthermore, the behavioural constructs discovered in this in study can be used to create a psychometric inventory to gauge the psychological polarity of T2D patients in terms of their commitment towards self-management behaviour.

*Comments from Reviewer 2*

1. Wat is the objective evidence that supports the first sentence in the conclusion?
   It is stated in the conclusion that "Evidence suggests that a more person-centered (instead of a disease-oriented) approach could break the deadlock impeding optimal disease control in T2D participants. In order to state this opinion, there should be the results comparing the difference between the efficacy of 'person-centered' approach and 'disease-oriented' approach. If there is the data representing the efficacy between these two different approaches, please describe those results in the manuscript to strengthen your conclusion.

- We thank the reviewer for pointing this out in the manuscript. We have taken steps to improve the clarity and to reflect the accuracy of our reporting of this statement as evidenced below.

Page 17, Lines 12-13:

An approach that prioritizes the needs of the patient (person-centered) rather than an exclusive focus on their “disease” alone could break the deadlock impeding optimal disease control in T2D patients[5].

2. Please add the references to the statements in order to make solid statements in the manuscript. For examples, the line 23-24 in page 9 saying "Generally, insulin users have positive view about using injectables and dismiss injecting insulin as being painful.", the line 11-12 in page 10 saying " With the passage of the time, the acceptance of the disease nurtures better medication compliance.", and the line 34-35 in page 10 saying "Patients believe insulin serves as a potent alternative to oral medication and clearly indicate a preference for insulin over oral medications." need references to support these statements.

- We believe there is a minor misunderstanding that could of lead to these comments. These sentences in the reflect the findings of our study (as evidenced by it’s place in the results section) and therefore do not require any citation. However, we appreciate the comments of the reviewer and took steps to ensure that this statement is not misread or misrepresented.

Page 9, Lines 13-24:

Generally, insulin users in our study have positive views about using injectables and dismiss injecting insulin as being painful.

Page 10, Lines 11-12:

With the passage of time, we found that patients’ acceptance of their disease cultivates better medication compliance.

Page 10, Lines 34-35:

Patients in our study believe insulin is a far potent alternative when compared to oral medication and clearly indicate greater preference for insulin over oral medications.

3. What are some new components being included compared to the previous conceptual framework in the figure 1, suggested by previous reports? In other words, please state more clearing what this study adds to current literature and current knowledge about this theme. Also, what are the relationship between the components presented in the figure 1? It is ambiguous to interpret the core message from the figure 1. Please clarify the conceptual framework more in detail.

- The conceptual model in Figure 1 depicts all the contructs that were generated from larger study within which the data from this study contributed to a part of the ‘internal environment’ of the framework called ‘disposition’. Similarly, in our recent published work in PLOS One (<https://journals.plos.org/plosone/article?id=10.1371/journal.pone.0225534>), we highlighted the volitional aspect of self-management practices which is another part of the larger model.
- Thus, in this manuscript, the author is attempting to introduce (not add) the personal dispositional aspects of the model (stated as positive and negative disposition in Figure 1), discussing it in detail what was discovered in the larger study.
- We have explained in the discussion secton what this model and the findings of this manuscript add to literature as discussed below.
- Additionally we have clarified the description of the conceptual model as shown below.

Page 7, Line 20 - 28

The internal psychological reality of T2D patients defining their self-management practices can be summarized into 2 main constructs – 1) positive disposition and 2) negative disposition. There appears to be a dynamic interaction between these constructs and numerous other concepts shown in Figure 1 – the factors associated with the external reality and the mediators of optimal behavior create the tapestry for the workings of the internal realities of T2D patients. The balance between an array of personal dispositional qualities appear to determine compliance with self-management practices (Figure 1, Table 3, Appendix A, Appendix B).

Page 16, Line 6 – 17:

This study depicts the summation of the various behavioural traits of the archetypal T2D people into one single operational model. Contrary to previous fragmentary descriptions of self-management practices in the form of systematic reviews or qualitative metanalysis[15–17], our study emphasizes on the connections and interactions that exist between these behavioural attributes of a typical T2D person found at a conventional clinical setting. While many of the themes presented in this study have been described elsewhere in literature[15–17], our study

collates these findings into a functional framework that define the attributes of T2D

patients with regards to self-management practices (Figure 1). Moreover, we have classified the psychosocial challenges of T2D patients into operational codes as a result of theory-building research techniques (Appendix A and B). This initiative is in tandem with the recommendations from the DAWN2 study which calls for the formulation of a distinct psychosocial approach that is specific to cultural sensitivities and societal norms[8].

4. For the strengths and limitations, I recommend to explain that the study is based on the Malaysian culture. This could be a strength if there are no other previous studies that investigated the T2D cases in Malaysia. Or, are authors suggesting the global common features of T2D? The authors should clarify and differentiate the Malaysia specific feature and global common feature.

- We thank the reviewer for this insightful comment. We have edited the manuscript accordingly.

Page 16, Line 6 – 10:

This study depicts the summation of the various behavioural traits of the archetypal T2D people into a unified operational model. Although many separate attempts were made to describe patient self-management behaviour in Malaysia, we uniquely examined and condensed of the overall behavioural attributes of T2D patients in Malaysia within single practice-based qualitative study[15–17]. Moreover, our findings widely corresponds with the results seen in other T2D patients assessed worldwide which further substantiates the opinion that most T2D patients share a common psychological feature in terms of the self-management behaviour[18–20].

5. Is there any specific reason why you presented the data in two different tables; table 1 and table 2? It would make a confusion to display demographic information in two different tables. Thus, I would recommend to combine the table 1 and table 2 to deliver the fundamental information in an effective way.

- We thanks the reviewer for this comment. The reason the authors resorted to divided this information was to display different demographic information about the 2 groups of participants in this study (T2D patients and healthcare professionals) in a more effective manner (and to avoid confusion). Furthermore, we would prefer to preserve the style, continuity and the coherence seen between this article and another published manuscript depicting another half of the same model .(<https://journals.plos.org/plosone/article?id=10.1371/journal.pone.0225534>).

6. There are so many grammatical errors such as misspelling, no space between two different words, plural with -s, and so on in the manuscript. Therefore, this manuscript definitely needs proofreading to correct those mistakes

Page 3, Line 2: "anda" -> "and a"
Page 7, Line 21: "managementwhich" -> management which"
Page 9, Line 3:"appearto"
page 15, line 24: appearsto implythat

- We would like to apologize for this technical error. These mistakes were not deliberate - there were compatibility issues between two different version of Microsoft Word that has lead to spacing errors. Many of these errors have been corrected as requested.
