# Supplementary material for: The internal realities of individuals with type 2 diabetes–Psychological disposition in self-management behaviour via grounded theory approach
Source: PLoS One. 2021 Apr 13;16(4):e0249620. doi: 10.1371/journal.pone.0249620 (PMC8043383; doi:10.1371/journal.pone.0249620)
Supplement: Attachment — Submitted filename: Comments.docx [file pone.0249620.s006.docx]

**Comments**

1. **Abstract: -** Page 8 line 2, you used an abbreviation entitled T2D. I tenaciously suggest you to rid abbreviations in the abstract part in all and when used threshold of the study.
2. **Key words: -** You used **non communicable diseases** as key word. I kindly connote you to use words and phrases that suggest what the topic is about.
3. **Introduction: -** Paragraph 2 line 4 you better write the full term than the abbreviation HbA1c. Analogously, DAWN-2 which is stated in paragraph 3, line 1. Because, they are abbreviations firstly written in the document.
4. **Design: -** Page 10 line 6, a word **socioeconomic** is meddled in a depart manner. I forward my perspective to use **sociodemographic** over **socioeconomic** throughout the document.
5. **Data collection: -** Page 13, first paragraph, line 1; you better not start with number. Also in this paragraph line 3, I suggest you to double for HCPs with HCP.
6. **Trustworthiness: -** Do u think that **analyzing and interpreting** the data by two investigators can made trustworthiness and is scientific? Think over the reason.
7. **References:** - You used too old references.
