# Supplementary material for: The internal realities of individuals with type 2 diabetes–Psychological disposition in self-management behaviour via grounded theory approach
Source: PLoS One. 2021 Apr 13;16(4):e0249620. doi: 10.1371/journal.pone.0249620 (PMC8043383; doi:10.1371/journal.pone.0249620)
Supplement: Attachment — Submitted filename: Response To Reviewers (7.2.2021).docx [file pone.0249620.s007.docx]

**Response to Reviewers**

We would like to thank the PLOS One for these suggestions. We also thank the reviewers for devoting their time to provide very insightful observations. We are certain this would definitely help strengthen the merit of our paper. Do not hesitate to keep us informed if further revisions are required.

*Comments from Academic Editor*

1. Please ensure that your manuscript meets PLOS ONE's style requirements, including those for file naming.

- We accept this suggestion and have made changes accordingly

2. Please include additional information regarding the survey or interview guide used in the study and ensure that you have provided sufficient details that others could replicate the analyses. For instance, if you developed a questionnaire as part of this study and it is not under a copyright more restrictive than CC-BY, please include a copy, in both the original language and English, as Supporting Information. In addition, please include further information concerning the development and/or pre-testing of this guide.

- Thank you for this important comment. We have included the requested topic guide and other files in the form of Supporting Information (S1 – S4)

3. Please provide the dates of patient recruitment to this study.

- Thank you for this important comment. We have integrated this piece of information into our manuscript.

Page 4, Lines 30 - 31:

The process of enrollment into this project took place between May 2018 to April 2019.

4. Please amend either the title on the online submission form (via Edit Submission) or the title in the manuscript so that they are identical.

- We accept this suggestion and have made changes accordingly

5. Please remove your figures from within your manuscript file, leaving only the individual TIFF/EPS image files, uploaded separately.  These will be automatically included in the reviewers’ PDF.

- We accept this suggestion and have made changes accordingly

6. Please include captions for your Supporting Information files at the end of your manuscript, and update any in-text citations to match accordingly. Please see our Supporting Information guidelines for more information: [http://journals.plos.org/plosone/s/supporting-information](about:blank).

- We accept this suggestion and have made changes accordingly

Page 20 - 21, Lines 33:

S1 Document. Topic guides for in-depth interviews and focused group discussions

S2 Table. Themes, Sub-Themes, Codes, Quotations within the Conceptual model (via Grounded Theory Approach) (DOCX)

S3 Document. Flow chart of the study design and development of conceptual framework (DOCX)

S4 Table. Consolidated criteria for reporting qualitative studies (COREQ): 32-item checklist. (DOCX)

*Additional Editor Comments*

1. Specific comments:
   Page 2, Line 6: In the abstract, “dimension” should be pluralized to “dimensions”
   Page 2, Line 7: In the abstract, “T2D patient” also needs to be plural, “patients”
   Page 2, Lines 16/17: Would recommend rephrasing the sentence so it does not start with a number. Perhaps something like this: “To examine the inner narratives about disease management and what they believe about the disease, 24 T2D patients and 10 healthcare professionals were recruited.”
   Page 2, Line 25: In the abstract results section, “to disease management” should be changed to “for disease…”
   Page 3, Lines 6/7: “2 folds” should be “2-fold” singular.
   Page 3, Line 13: HCP should be plural (HCPs) when referring to more than one HCP.
   Continued minor edits (e.g., plural vs singular, missing words, extra words) evident and need to be addressed.

Page 3, Line 28: Grounded should not be capitalized.

- We accept this suggestion and have made changes accordingly

2. Introduction:
   Page 3, Lines 26-28: Would suggest not talking about publishing the procedures, but the aim of the paper is sharing the results related to…using the protocol previously published (11).

Page 3, Line 34 – 35

This paper aims to expound on the self-management behaviour in people with T2D closely related to an integrated behavioural model proposed in a previously published protocol[11]

3. Methods:
   Pages 5-6, Tables 1 and 2: I am concerned about the confidentiality of data when there are cells that have less than 5 participants. These may be identifiable. Please revise, could use <5 for those cells, or report in more broad categories.
   Page 7, Line 5: This paper uses a lot of abbreviations and acronyms. I would suggest not abbreviating in-depth interviews and removing the IDI abbreviation. It is not commonly used. Please attend to these revisions throughout the paper.

- We accept this suggestion and have made changes accordingly

4. Data Collection and Analysis:
   Reviewer 2 suggests adding information on constant comparative analysis as this is key to grounded theory. I would agree, as it currently stands, your methods could easily reflect a descriptive qualitative study.

- We accept this suggestion and have made changes accordingly

Page 8, Line 19

Both interviewing technique and the topic guide utilized in this study is described in a published research protocol[11]. After the 10^th^ round of interviews, the interviews did not uncover newer themes and theoretical saturation was considered to have been achieved. The validity of the emergent narratives from these themes was explored, reinforced, and finalized via additional interviews with 14 individuals with T2D.

5. Discussion:
   The discussion section is very short and provides only a summary of the results as it currently appears. I would put the “comparison to literature” section after the discussion summary, or perhaps amalgamate these sections and then present strengths and limitations. Authors also need to discuss the impacts/relevancy for policy and practice. What is needed is the “So what?”

- Thank you for this valuable advice, we concur and have made changes accordingly

Page 21, Line 12 - 27

Our research initiative supports the need to transform naturalistic narratives derived from qualitative inquiry into measurable scales that accurately reflect the behaviour inherent to the population that is being studied[28]. This carefully curated real-world data and the subsequent process of interpretative deduction have uncovered practical targets for behavioural change that can be easily integrated into routine clinical practice. This step (as opposed to standard care alone) could facilitate a more person-centered approach, leading to smoother behavioural transformation in people with T2D

This study's overarching objective is to create a realistic benchmark for self-management behaviour in T2D people from data that is acquired from a naturalistic environment[29]. Many pre-existing scales in literature have not been rigorously evaluated in terms of validity and reliability[29]. Furthermore, these inventories lack robust psychometric properties, rendering it challenging to detect any changes in behaviour as a result of behavioural change intervention[29]. To that end, the creation of this operational model for self-management behaviour can ultimately generate a largely cohesive psychometric inventory explaining the many different shades of emotions that characterize the psyche of individuals with T2D.

6. Strengths and Limitations:
   Some of the information included here may be able to be highlighted/moved to the discussion section.
   I also find that all of the results are heavily focused on medications and use of the same. Self-management is a much bigger topic than only medications. It should include eating habits/nutrition, exercise, and mental health and wellbeing, the latter which is addressed somewhat. I would encourage that if there is data, to more fully report the results. If there is no data, perhaps this is a study of self-management of medications and some of the wording and title may need to reflect that. You may also want to delineate this as a limitation in your limitations section

- Thank you for this important comment. We have included more quotations into the manuscript to reflect other aspects of self-management behaviour (exercise and diet)

Page 15, Line 8 - 9

“I couldn’t accept reality [diagnosed with T2D]. I felt I could just go on with my life as usual. I made sure I did more exercise. I still indulged in food”. (52, Male)

Page 12, Line 28 – 37:

As far as lifestyle modification is concerned, most T2D people find the adherence to dietary restrictions burdensome only during at the initial phases of their disease. Moreover, they believe these dietary measures are critical to the long-term control of T2D.

“Initially, when I was told to control my diet, I thought it was missing it all [food]. But that was only in the beginning. Soon, I became accustomed to it.” (60, Male)

“You see, everything you consume will affect your sugar levels. You have to understand that. So there is a limit to everything. If you don’t control it [food], your sugar levels will increase”. (52, Male)

Page 13, Line 27 – 35:

Additionally, individuals with T2D are highly conscious of the need for adequate physical exercise and how it could potentially help achieve better control of their disease. Many T2D people believe that exercising can work as an adjunct in lowering blood glucose levels in their body.

“It doesn’t matter what my sugar levels might be, I will do exercise and look after my diet so that I will not experience any complications” (80, Male)

“I love to exercise, it will bring down sugar levels in our body” (65, Female)

7. References:
   Reviewer 1 discusses the need for more updated references. I would agree that some of the references are fairly old, particularly those that provide statistics, or other components of self-management. We do understand that there are some seminal references, particularly around analysis and design. Please review and revise the references accordingly.

- Thank you for this comment. All references were updated to include publications from year 2000 and beyond.

*Comments from Reviewer 1*

1. Abstract: - Page 8 line 2, you used an abbreviation entitled T2D. I tenaciously suggest you to rid abbreviations in the abstract part in all and when used threshold of the study.
8. We accept this suggestion and have made changes accordingly
2. Key words: - You used **non communicable diseases** as key word. I kindly connote you to use words and phrases that suggest what the topic is about.
9. We accept this suggestion and have made changes accordingly
3. Introduction: - Paragraph 2 line 4 you better write the full term than the abbreviation HbA1c. Analogously, DAWN-2 which is stated in paragraph 3, line 1. Because, they are abbreviations firstly written in the document.
10. We accept this suggestion and have made changes accordingly
4. Design: - Page 10 line 6, a word socioeconomic is meddled in a depart manner. I forward my perspective to use sociodemographic over socioeconomic throughout the document.
11. We accept this suggestion and have made changes accordingly
5. Data collection: - Page 13, first paragraph, line 1; you better not start with number. Also in this paragraph line 3, I suggest you to double for HCPs with HCP.
12. We accept this suggestion and have made changes accordingly
6. Trustworthiness: - Do u think that analyzing and interpreting the data by two investigators can made trustworthiness and is scientific? Think over the reason.

- Thank you for this insightful comment. Allow us to clarify this statement. The primary codes for the transcript was coded and conceptualized by the main researcher (YSN). The use of independent coders was meant to verify or substantiate the stability of the codes established by the main researcher. We have edited the manuscript to provide a clearer description of this process.

Page 8, Lines 7 - 10:

All transcripts were coded independently. A list of emerging themes and categories was thengenerated by the primary researcher (YSN). Subsequently, two independent coders scrutinized and coded the transcripts for categories, themes, and sub-themes. An excellent inter-coder consistency was found (Cohen’s κ =0.84).

7. References: - You used too old references.

- Thank you for this comment. All references were updated to include publications from year 2000 and beyond.

*Comments from Reviewer 2*

1. I have gone through your document and I got it very good and sound able. I strongly suggest you the authors collect and then aggregate comments I have mentioned on the recommendation paper. Hoping you are proofreading the document so that a lot of grammatic and spelling disorganizations are modified.

- We accept this suggestion and have made changes accordingly

*Comments from Reviewer 3*

1. Even though the authors adequately explained which purposive sampling technique did they used, they did not mention anything about which criteria/rule of saturation have they used; in order to know when to stop further data collection and start to count the number of people who had participated and eventually know the final sample size. Hence, I would like to ask the authors when was the point where they feel reasonably confident that they have saturated a particular source of information to the point of redundancy or when was the point where they feel that the incoming data have adequately answered their research questions?

- Thank you for this valuable advice, we concur and have made changes accordingly

Page 8, Line 19

Both interviewing technique and the topic guide utilized in this study is described in a published research protocol[11]. After the 10^th^ round of interviews, the interviews did not uncover newer themes and theoretical saturation was considered to have been achieved. The validity of the emergent narratives from these themes was explored, reinforced, and finalized via additional interviews with 14 individuals with T2D.

2. page 7, line 4--> “… Ultimately, 24 patients and 10 HCPs took part in in-depth interviews (IDI) conducted between…”

   -->the stated data collection technique that the authors used for the 10 HCPs should not be named an ‘in-depth interview’. Because, the HCPs were not being interviewed about themselves (they are not a diabetic patients). In-depth interview is all about the participants themselves. Therefore, I would recommend it be changed to a ‘key informant interview’, as they have been interviewed only because of their position or due to the fact that they are well aware about the disease instead of being patients.

- We accept this suggestion and have made changes accordingly

Page 7, Line 7 - 8

Ultimately, 24 people with T2D (in-depth interviews) and 10 HCPs (key-informant interviews) took part in this study

3. I wonder how the authors decided on how many focus groups to form? it is generally recommended to conduct at least two FGDs for a certain defining demographic variable. Was it conducted based on gender? Having complication or not? any other variables? Or by simply dividing the number of patients in to two randomly?

- Thank you for this comment. The focus group discussion was conducted to collectively confirm the narratives and the codes obtained from the initial coding after the in-depth interviews. This step helped establish the robustness and the validity of our data (data triangulation). The participants of each FGD session were chosen based upon similarities in ideas and codes that were generated throughout the coding process.

4. On the data collection, data management, and analysis section, I recommend mentioning the use of constant comparative analysis. As the research is done via grounded theory, it is basically expected to do simultaneous collection and analysis of the data.

- We thank the author for this insightful comment. We have edited the manuscript to show a clearer picture of this processes. Moreover, we have included Supporting Files 1 – 4 which should the iterative process undertaken, evidenced by the evolution in the topic guides used and the emergent nature of the data that were obtained.

Page 7, Line 12

All participants were interviewed in a room within the confines of the clinic that was allotted specifically for the purpose of this study. In-depth interviews were guided by research questions (compiled in the form of a topic guide; S1 Document) designed to assess the inherent psychological attributes that are closely associated with self-management behaviour in persons with T2D[11]. Steps were also taken to ensure these interviews remain unstructured enough to allow the discovery of novel concepts and ideas. Moreover, the topic guide was constantly updated in tandem with the acquisition of newer themes from these interviews. Both interviewing technique and the topic guide utilized in this study is described in a published research protocol[11]. After the 10^th^ round of interviews, the interviews did not uncover newer themes and theoretical saturation was considered to have been achieved. The validity of the emergent narratives from these themes was explored, reinforced, and finalized via additional interviews with 14 individuals with T2D. The interviews were audiotaped and transcribed verbatim. Field notes were made in the form of memoing during and after the interviews. One researcher (YSN) conducted all interviews.

5. “There is no full form of the abbreviation “IDM” in the verbatim. The verbatim needs to be given demographic details like for example: “35-year-old T2D, self-employed male, FGD participant””.
However, I could not find this change in the post comment Manuscript Draft. I apologize if I missed this or if I am having the pre comment Manuscript Draft. Can the authors please double check to confirm they included this information?

- Thank you for this comment. The abbreviation has been removed from the entire manuscript.

6. page 9, line 14--> this should be “…indicates that most…” rather than “…indicate that most...”
 page 11, Line 11--> Remove “the” before “… taking medication in accordance….”
 page 11, Line 26--> add “of” after “… People with diabetes recognize the importance….”
 page 19, line 4--> there is no space between two the words “….implementand appraise…”
 page 7, line 4--> This should be “….in the in-depth interviews (IDI) conducted…”

- We accept this suggestion and have made changes accordingly
