# Supplementary material for: The internal realities of individuals with type 2 diabetes–Psychological disposition in self-management behaviour via grounded theory approach
Source: PLoS One. 2021 Apr 13;16(4):e0249620. doi: 10.1371/journal.pone.0249620 (PMC8043383; doi:10.1371/journal.pone.0249620)
Supplement: Attachment — Submitted filename: Response To Reviewers (27.2.2021).docx [file pone.0249620.s008.docx]

**Response to Reviewers**

We would like to thank the PLOS One for an excellent peer review and editorial process. Below are our responses to the comments from reviewers.

*Comments from Academic Editor*

1. There are many lines in the manuscript that only have a word or two. The formatting needs to be adjusted.

- Thank you for your comment, we have amended the manuscript accordingly.

2. There are many specific comments that still require they be addressed. They are listed as follows:

   • Short title in your manuscript still does not match that in the introductory form
   • Page 2, second and ninth lines, results section, and conclusion section of the abstract please spell out type 2 diabetes or consistently use the abbreviation.
   • Page 2, methods in abstract should say “a” grounded theory approach instead of “the.” Also would suggest not capitalizing grounded theory.
   • Page 2, second line of methods in abstract remove T2D
   • Page 2, last line of results should read motivational “and” follow through
   • Page 4, second line under design has an extra bracket in the middle of the line.
   • Page 4, 3rd line in the design section would consider using “individuals” instead of people.
   • Page 4, suggest changing “good spread” to diverse sample.
   • Page 5, Line 6, “comprising” should be changed to “comprised”
   • Tables 1 and 2 still have some cells with less than 5 participants (e.g., ethnicity – Chinese – 1) Please revise accordingly.
   • Page 7, top of the page, numbers reported on number of participants should go before Tables and with the demographic data.
   • Bottom of page 7, “remain” needs to be “remained”
   • Bottom of page 7, top of page 8: the topic guide you have already talked about and referenced as reference 11. Suggest adding the interview technique above as well so this isn’t repeated.
   • Text at bottom of page 7 and top of page 8 is in a different font.
   • Page 8 – not sure if trustworthiness can be made certain; it can certainly be enhanced and facilitated.
   • Page 13, first paragraph, “They believe that it is necessary to consume medications lifelong…” Reword as “They believe that it is necessary for lifelong medications…” for clarity and flow.
   • Page 15, second last paragraph, “appear” should be “appears.”
   • Page 21, paragraph two, “we emphasized on inclusivity…”, remove “on.”
   • Acknowledgements should have the “e’ in the word. Please replace the same.

- Thank you for these comments, we have amended the manuscript accordingly.

3. Page 8 – why were two qualitative software packages used?

- We accept this suggestion and have made changes accordingly

Page 7, Line 27 - 31

(Atlas.TI qualitative analysis program, Version 7, Cincorn Systems Inc, 2008 [coding of transcripts and generation of themes]; NVivo Plus, Version 12, QSRInternational, 2019 [to determine inter-coder and content consistency])…

4. Page 8 – analysis still does not contain a lot of information on grounded theory approach (e.g., constant comparative analysis).

- We accept this suggestion and have made changes accordingly. We have included S2 Figure as a part of the Supplemental Files in this submission to outline these processes in detail. We have also made changes to the text as follows:

Page 7, Lines 16 - 18:

Both interviewing technique and the topic guide utilized in this study are described in a published research protocol[11]. A detailed description of this process is summarized in a flowchart in S2 Figure.

5. Results section – you have changed much of language to people with T2D. This is a generalization and your results only apply to your participants. You will need to address and make appropriate changes.

- We concur and made changes to the manuscript by specifying the exact participants of this study by using terms such as “T2D participants”/”type 2 diabetes individuals in our study”.

6. Page 12 – two thirds of the way down, “only during at the initial phases of their disease.” This phrase needs to be revised.

- We accept this suggestion and have made changes accordingly

Page 11, Line 34 – 36

As far as lifestyle modification is concerned, most T2D participants find the adherence to dietary restrictions burdensome only at the preliminary phase of their disease.
