# Supplementary material for: The internal realities of individuals with type 2 diabetes–Psychological disposition in self-management behaviour via grounded theory approach
Source: PLoS One. 2021 Apr 13;16(4):e0249620. doi: 10.1371/journal.pone.0249620 (PMC8043383; doi:10.1371/journal.pone.0249620)
Supplement: Attachment — Submitted filename: Response To Reviewers (17.3.2021).docx [file pone.0249620.s009.docx]

**Response to Reviewers**

We would like to thank the PLOS One for an excellent peer review and editorial process. We apologize for not being thorough with our previous revision process. In this current submission, we have taken every step possible to ensure that all comments have been addressed as stated below. Please let us know if additional revisions are required.

*Comments from Academic Editor*

1. Tables 1 and 2 still have cells with less than 5 participants. Please review and mark all those that are under 5 as <5.

- Thank you for your comment, we have amended the manuscript accordingly.

2. There are still some areas with extra space and lines. Please remove and ensure your manuscript is consistent throughout. Also there are still a few lines that have only a few words and then go to the next line. Please remove the same

- Thank you for your comment, we have amended the manuscript accordingly.

3. Response and actions, if appropriate, to the comment re: retracted references.

- Thank you for this comment. In line with previous comments (outdated references), we have removed 3 citations below from the main text:
- Lau RR, Hartman KA. Common sense representations of common illnesses. Heal Psychol. 1983;2: 167–185.
- Leventhal H, Meyer D ND. The common sense representation of illness danger. Med Psychol. 1980;2: 7–30.
- Leventhal H, Weinman J, Leventhal EA, Phillips LA. Health psychology: the search for pathways between behaviour and health. Annu Rev Psychol. 2007;59: 1–9.
- These citations have been effectively subsumed (preserving factual content and accuracy) into a single reference:
- Ogden J. Health psychology - a textbook. 2012th ed. Berkshire: Open University Press; 2012.
- Hence, the reference section has been redacted from 30 citations (manuscript labelled 23.1.2021) to 27 in the current form.
